# Alinity m, a Random-Access System, for Hepatitis B Virus DNA Quantification in Plasma and Whole Blood Collected on Dried Blood Spots

**DOI:** 10.1128/msphere.00082-22

**Published:** 2022-04-28

**Authors:** Valérie Ortonne, Quentin Lucas, Olivia Garrigou, Alexandre Soulier, Dominique Challine, Jean-Michel Pawlotsky, Vincent Leroy, Stéphane Chevaliez

**Affiliations:** a National Reference Center for Viral Hepatitis B, C and Delta, Department of Virology, Hôpital Henri Mondor, Créteil, France; b Department of Hepatology, Hôpital Henri Mondor, Créteil, France; c INSERM U955, Créteil, France; University of Michigan-Ann Arbor

**Keywords:** hepatitis B virus, HBV DNA, real-time PCR, whole blood, dried blood spot

## Abstract

The International Liver Association recommends the use of accurate and sensitive molecular methods for determination of hepatitis B virus (HBV) DNA levels in plasma or serum of chronic HBsAg carriers. The level of HBV replication represents the strongest predictive biomarker associated with disease progression and long-term outcome of chronic HBV infection. The purpose of this study was to evaluate the ability to the new Alinity m System to detect and quantify HBV DNA in plasma and whole blood collected on dried blood spots (DBS). Paired plasma and DBS samples from patients chronically infected with various HBV genotypes were tested in parallel for HBV DNA detection and quantification. There is a linear relationship between HBV DNA levels measured in plasma samples using the Alinity m HBV assay and the Xpert HBV viral load assay, used for comparison. A slight deviation (0.03 ± 0.31 log IU/mL) was observed within the quantitative range. In DBS, HBV DNA levels closely correlated with levels measured in plasma. All patients had detectable and quantifiable HBV DNA by DBS testing, except for one patient with a plasma HBV DNA level above 2,000 IU/mL. In conclusion, the newly developed real-time PCR-based assay Alinity m HBV assay can correctly detect HBV DNA in DBS, especially for patients with blood HBV DNA levels above 2,000 IU/mL, and also accurately quantify HBV DNA in plasma samples.

**IMPORTANCE** Hepatitis B virus is one of the most prevalent blood-borne viruses affecting the liver and causing acute and chronic hepatitis. Only a small proportion of people with HBV infection are diagnosed. HBV DNA measurement is critical in clinical practice for the diagnosis and treatment decisions of patients requiring antiviral therapy. Dried blood spot (DBS) collection provides a simple, practical, and acceptable alternative to venous blood collection, especially in community settings. We have demonstrated high sensitivity and specificity for HBV DNA detection in DBS compared to plasma samples, especially when using clinically relevant cutoffs of 2,000 and 20,000 IU/mL. Results support the use of DBS in community-based settings.

## INTRODUCTION

Hepatitis B virus (HBV) infection is one of the major public health problems worldwide. In 2019, an estimated 296 million people were chronically infected with HBV globally, with the highest burden of chronic hepatitis B in low-middle-income countries (1). Chronic HBV infection can lead to cirrhosis and hepatocellular carcinoma (HCC). Liver complications cause an estimated 820,000 deaths annually (1). Only a small proportion of people with hepatitis B virus infection are diagnosed (2). In 2016, the World Health Organization (WHO) estimated that 27 million (10.5%) of hepatitis B surface antigen (HBsAg)-positive carriers were diagnosed and 4.5 million (16.7%) were treated (3). Therefore, expanding diagnostic access to improve treatment eligibility is critical. Well-tolerated orally potent antiviral drugs with high barriers to resistance (i.e., entecavir and tenofovir) have been available since 2006 and have been shown to be highly effective in inhibiting viral replication in the long term and preventing liver disease progression.

Measurement of HBV DNA level in serum or plasma is essential for diagnosis, determining the phase of infection (particularly in individuals with HBeAg-negative chronic infection [inactive carrier]), making a treatment decision and subsequent monitoring of patients in the assessment of the efficacy of antiviral treatment, and, finally, monitoring viral resistance (4, 5). Accurate diagnosis of HBV infection is the key and represents the entry point into the cascade of care for infected patients.

Most HBV DNA assays and platforms used in clinical practice utilize real-time PCR technology with a sensitivity of at least 10 international units (IU) per milliliter and a broader range of linear quantification, up to 9 log IU/mL for newer assays. Other techniques, including transcription-mediated amplification-based assays and isothermal amplification-based assays, are also available for HBV DNA detection and quantification (6, 7). The cobas AmpliPrep/cobas TaqMan HBV test version 2.0 (Roche Molecular Systems) and Abbott RealTime HBV assay (Abbott Molecular) were the most widely used assays over the last decade. Although they demonstrated adequate analytical performance for HBV DNA detection and quantification, delivery of the first results required a long period of time (up to 5 to 6 h). As a result, advances have been made with the elimination of batch processing of samples and automation of all parts of nucleic acid testing, allowing achievement of the expected results in less than 2 h for the more recent platforms and assays. Although none of the commercial HBV DNA assays are U.S. Food and Drug Administration (FDA) approved, Conformité Européene (CE) marked, and/or WHO prequalified for whole blood recovered from dried blood spots (DBS), the WHO recommends the use of DBS specimens to test HBV DNA in specific settings where nucleic acid testing is unavailable and/or for people with poor venous access (1). Dried blood spot specimens provide an alternative to serum and plasma for diagnosis and monitoring of patients infected with HBV (8–10). DBS specimens are simple to collect, process, and package and can be shipped at ambient temperature. Several studies comparing HBV DNA levels in plasma or serum and in DBS have been conducted but used various methodologies, highlighting a large heterogeneity in DBS preparation and elution protocols and making comparison between studies problematic. Most of the individual studies have shown a significant underestimation of HBV DNA levels compared to standard matrices, 1 to 2 log IU/mL lower, but suggest that the sensitivity of HBV DNA detection using DBS above clinically relevant cutoffs of 2,000 or 20,000 IU/mL is satisfactory (11). That means that testing using DBS specimens will be able to identify the majority of persons with chronic hepatitis in need of antiviral treatment according to the international recommendations of clinical practice on the management of HBV infection (4, 5).

In this study, we examined the performance of the Alinity m System, a fully automated, continuous, random-access molecular diagnostic analyzer, to detect and quantify HBV DNA in DBS and plasma specimens from patients infected with various HBV genotypes.

## RESULTS

### HBV DNA detection and quantification in plasma specimens.

To study the effect of the HBV genotype on HBV DNA quantification, plasma samples from HBsAg-positive patients infected with various HBV genotypes (A to E and G) were tested in parallel with the Alinity m HBV and Xpert HBV viral load assays.

**FIG 1:**
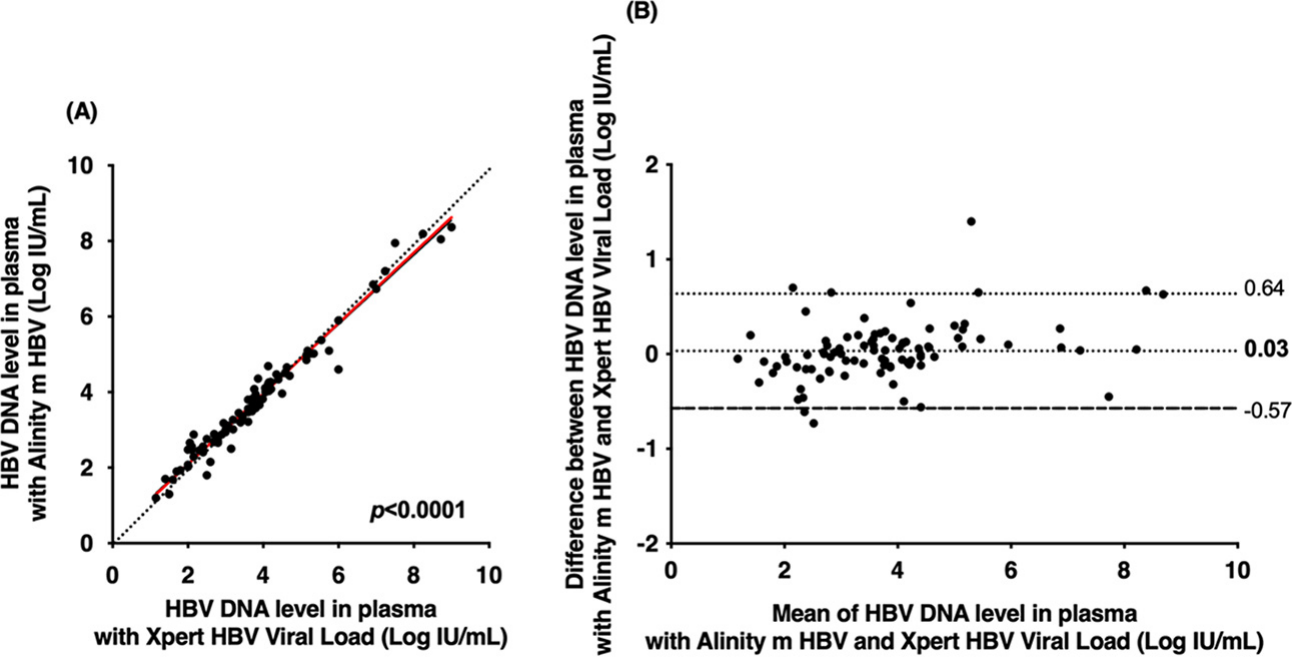
(A) Deming regression of HBV DNA levels measured by the Alinity m HBV assay and Xpert HBV viral load assay in 91 plasma specimens containing HBV genotype A (*n* = 14), B (*n* = 7), C (*n* = 4), D (*n* = 9), E (*n* = 39), or G (*n* = 3) or an unidentified genotype (15 cases). The red solid line represents Deming regression, and the black dotted line represents the reference (i.e., no difference between the two techniques). (B) Bland-Altman plot analysis of HBV DNA levels measured by the Alinity m HBV assay and Xpert HBV viral load assay in 91 plasma specimens containing HBV genotype A (*n* = 14), B (*n* = 7), C (*n* = 4), D (*n* = 9), E (*n* = 39), or G (*n* = 3) or an unidentified genotype (15 cases). The black dotted and dashed lines represent deviation ± 1.96 SD, respectively.

Of the 138 samples, 91 belonged to the dynamic range of quantification of the two assays. Of the 9 samples that were quantified by the Alinity m HBV assay (mean HBV DNA level of 1.50 ± 0.20 log IU/mL), 6 were found to be below the lower limit of quantification (<1.0 log IU/mL) and 3 were not detected by the Xpert HBV viral load assay. For the remaining 38 plasma samples, neither assay detected HBV DNA. Figure 1A shows the relationships between the HBV DNA concentrations measured by the two assays. A strong correlation was found (*r* = 0.982; Deming regression equation, *y* = 0.934*x* + 0.22). In the Bland-Altman analysis, slight differences were observed within the quantitative range of the two assays (0.03 ± 0.31 log IU/mL). No quantitative differences in genotype or HBV DNA level were observed (Fig. 1B). For 92.3% (84/91) of plasma specimens, the difference between assays was within 1.96 times the standard deviation (SD) of bias. For the remaining 7.7% (7/91) of specimens, the difference exceeded 1.96 times the SD of bias with the Alinity m HBV assay compared to the Xpert HBV viral load assay: 5.5% (5/91) of the results were higher, while 2.2% (2/94) were lower.

**FIG 2:**
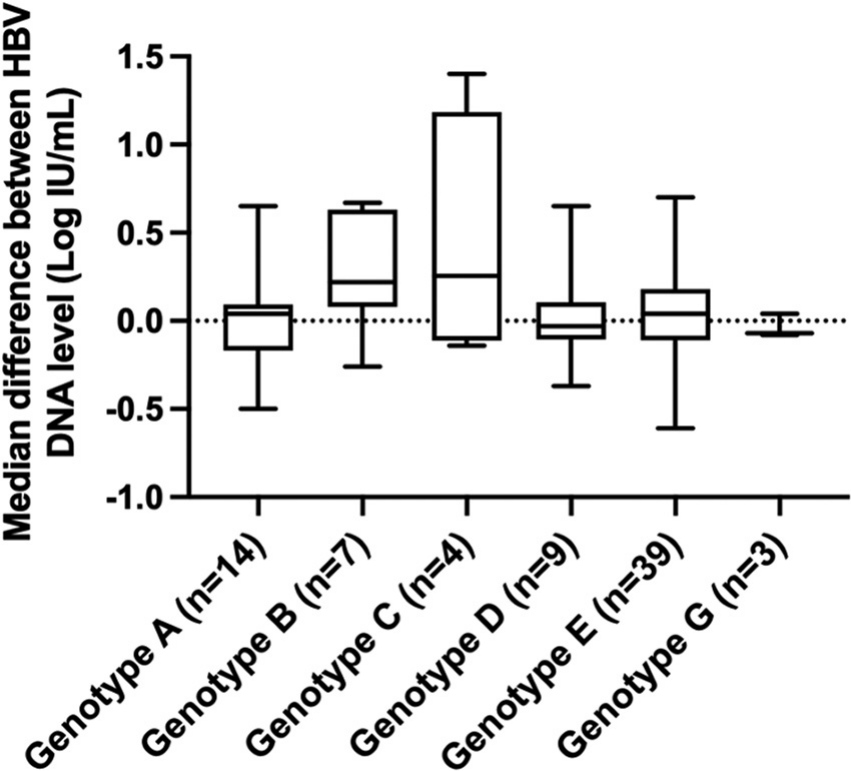
Box plot representation of the distribution of the differences between HBV DNA levels determined by the Alinity m HBV assay and the Xpert HBV viral load assay according to HBV genotype. The midline and the lower and upper edges of boxes represent the median value, 25th percentile, and 75th percentile, respectively. The lower and upper error bars represent the minimum and maximum values, respectively.

Figure 2 shows individual difference between the Alinity m HBV and Xpert HBV viral load assays for each genotype. That confirms that HBV DNA levels were generally overestimated in the Alinity m HBV assay compared to the Xpert HBV viral load assay, with the exception of genotypes D and G (Fig. 2).

### Overall percent agreement for the clinical breakpoints at 2,000 and 20,000 IU/mL.

Table 1 shows the overall agreement of the Alinity m HBV and Xpert HBV viral load assays for the two clinically relevant cutoffs of 2,000 and 20,000 IU/mL in plasma samples. At the 2,000 IU/mL cutoff, the overall agreement was 98.9% (90/91). At the 20,000 IU/mL cutoff, the overall agreement between the two assays was slightly lower, at 96.7% (88/91).

**TABLE 1:** Concordance at the 2,000 and 20,000 IU/mL clinically relevant cutoffs between the Alinity m HBV and Xpert HBV viral load assays (number of samples)

Alinity m HBV	Xpert HBV viral load		
	<2,000 IU/mL	≥2,000 IU/mL	Total
<2,000 IU/mL	35	1	36
≥2,000 IU/mL	0	55	55
Total	35	56	91
	<20,000 IU/mL	≥20,000 IU/mL	Total
<20,000 IU/mL	66	1	67
≥20,000 IU/mL	2	22	24
Total	68	23	91

### HBV DNA detection and quantification in DBS.

**FIG 3:**
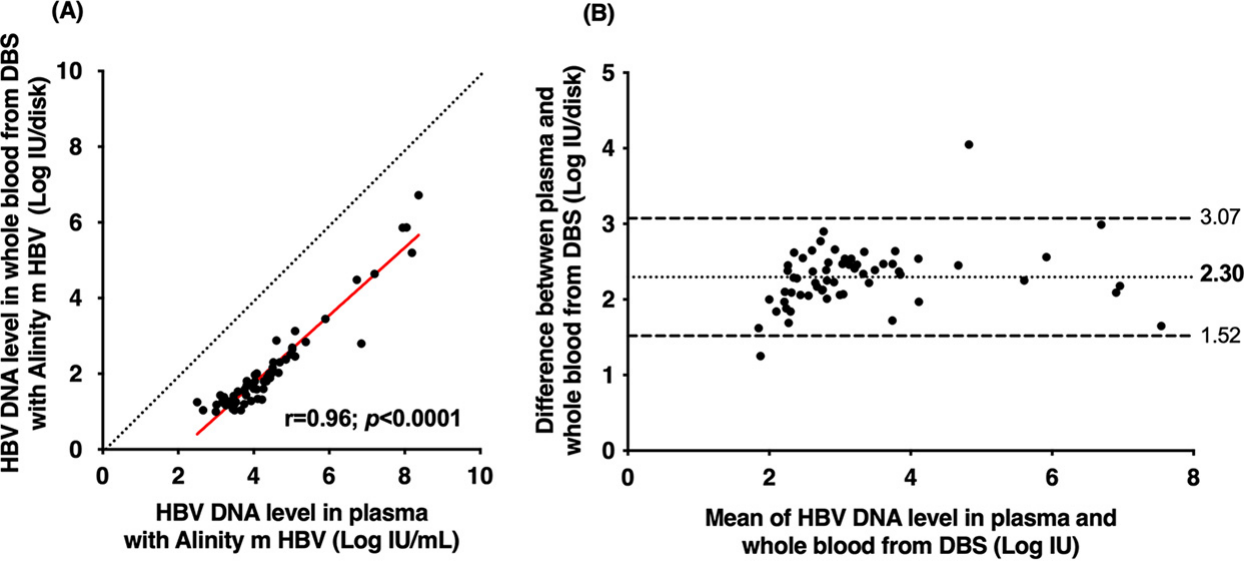
(A) Linear regression of HBV DNA levels measured by Alinity m HBV assay in 61 paired plasma and DBS specimens containing HBV genotype A (*n* = 10), B (*n* = 6), C (*n* = 1), D (*n* = 6), E (*n* = 33), or G (*n* = 1) or an unidentified genotype (4 cases). The red solid line represents the linear regression and the black dotted line represents the reference (i.e., no difference between plasma and DBS specimens). (B) Bland-Altman plot analysis of HBV DNA levels measured by the Alinity m HBV assay in 61 paired plasma and DBS specimens containing HBV genotype A (*n* = 10), B (*n* = 6), C (*n* = 1), D (*n* = 6), E (*n* = 33), or G (*n* = 1) or an unidentified genotype (4 cases). The black dotted and dashed lines represent deviation ± 1.96 SD, respectively.

To evaluate the use of DBS specimens as a tool to identify patients with ongoing HBV infection, paired plasma and venous whole blood spotted onto filter paper (DBS specimens) from HBsAg-positive patients were tested in parallel with the Alinity m HBV assay running on the Alinity m System. Among 138 patients with chronic HBV infection, 130 underwent HBV DNA testing in DBS. The remaining 8 patients had no DBS sample available. The overall sensitivity of the Alinity m HBV assay for the HBV DNA detection in DBS specimens was 78.1% (95% confidence interval [CI], 68.9% to 85.2%), and the specificity was 100% (95% CI, 89.9% to 100%) (Table S1). Twenty-one DBS samples demonstrated discrepant results: HBV DNA was not detected in DBS, while it was detected and quantified in plasma by the Alinity m HBV assay. Most of them had a low HBV DNA level in plasma, with a median of 1.80 log IU/mL. Among them, 13 patients had an HBV DNA level of <2 log IU/mL, 7 had an HBV DNA level between 2 and 3 log IU/mL, and only 1 patient had an HBV DNA level higher than 3 log IU/mL (i.e., 3.6 log IU/mL). The sensitivities for HBV DNA levels of >2 log IU/mL and >3 log IU/mL were 92.7% (95% CI, 85.7% to 96.4%) and 99.0% (95% CI, 94.3% to 99.8%), respectively. Using the Alinity m HBV assay, 61 patients were found to have quantifiable HBV DNA in DBS samples. A significant positive correlation was observed between HBV DNA concentrations measured in paired DBS and plasma samples independent of HBV DNA concentration and genotype (*r* = 0.96; *p* < 0.0001) (Fig. 3A). A deviation of 2.30 log IU was observed between HBV DNA levels measured in plasma and DBS as shown by Bland-Altman plot analysis. The HBV DNA level in plasma and DBS differed by 1.96 times the SD of bias in only 2 samples, including a higher genotype A sample and lower genotype D sample (Fig. 3B).

10.1128/msphere.00082-22.1 TABLE S1 Sensitivity and specificity of the Alinity m HBV assay for HBV DNA detection in whole blood collected on DBS compared to plasma Download Table S1, DOCX file, 0.01 MB. Copyright © 2022 Ortonne et al. 2022 Ortonne et al. https://creativecommons.org/licenses/by/4.0/ This content is distributed under the terms of the Creative Commons Attribution 4.0 International license.

All patients had detectable and quantifiable HBV DNA by DBS testing, except for one patient with a plasma HBV DNA level above 2,000 IU/mL. In contrast, only 8 of the 43 patients with an HBV DNA below 2,000 IU/mL in blood had HBV DNA detectable and quantifiable by DBS testing.

## DISCUSSION

International clinical practice guidelines recommend sensitive and accurate nucleic acid amplification techniques for detection and quantification of HBV DNA in clinical practice (4, 5). Measurement of HBV DNA level is critical for ongoing HBV infection diagnosis, predicting the prognosis of liver disease, guiding treatment decisions, monitoring of virological response to antiviral therapy, and the emergence of resistance. Dried blood spot technology has become a practical tool for qualitative and quantitative biological analyses, enabling the analysis of multiple analytes such as viruses, nucleic acid, antibodies, and antigens in a single sample. Whole blood collected on nitrocellulose filer paper greatly improved blood sample collection in resource-constrained settings. The use of DBS in virus screening and nucleic acid detection by real-time molecular techniques has received increasing attention. Standardized quality control methods for DBS can be used to monitor antiretroviral therapy, diagnose, and identify genetic signatures associated with HIV-1 infection by PCR. Hepatitis B virus serological and nucleic acid testing from DBS specimens has been successfully performed in clinical trials, epidemiological surveillance programs, screening programs, and limited clinical setting. However, there are currently no registered assays for HBV DNA testing in DBS samples.

The results of this study demonstrate that the new Alinity m HBV assay can accurately quantify HBV DNA level in plasma specimens, as previously described (12). Its performance is consistent with those of other real-time platforms and assays commonly used in clinical practice (8, 9, 12–15). An excellent correlation (correlation coefficient ≥ 0.98) was observed between the Alinity m HBV and Xpert HBV viral load assays. The Alinity m HBV assay showed only minor differences in HBV DNA levels from those obtained with the Xpert HBV viral load comparator assay, with a deviation of 0.03 log IU/mL, and 98.9% of quantifiable patient results had a difference of <1 log IU/mL. Differences in performance were independent of HBV DNA concentration and HBV genotype and are likely not to affect clinical practice. In addition, agreement between the two assays was generally high across key clinical decision points, including the 2,000 IU/mL and 20,000 IU/mL thresholds. Not surprisingly, the risk of conflicting results increases as HBV DNA levels approach the 2,000-IU/mL or the 20,000-IU/mL threshold, as previously documented with other platforms and assays (16, 17).

To our knowledge, this is the first published study demonstrating that DBS collection and testing on the Alinity m System are acceptable and feasible for implementation in a simplified testing pathway in medical facilities where venipuncture is difficult and/or where laboratory services are unavailable. In our study, DBS could be safely used to determine active hepatitis B virus infection in patients with HBV DNA levels above 3 log IU/mL, using standardized commercially available methods, although molecular platforms and assays are not available for DBS testing (11, 18, 19). In this case, using the Alinity m HBV DNA assay increased the sensitivity of HBV DNA detection from DBS to 99%. Molecular testing using DBS sampling will be able to identify the majority of HBeAg-negative patients with chronic infection or chronic hepatitis who are candidates for antiviral therapy (4).

There are some limitations to this study. First, the remnant DBS samples were prepared in the laboratory by spotting venous whole blood from clinical samples onto cellulose filter paper, rather than being collected from the patient fingerstick. The sample set was small, with low to moderate concentrations (average HBV DNA level of 3.60 log IU/mL), and only 78 of 100 patients with detectable HBV DNA were genotyped. HBV DNA detection and quantification were performed on only one spot. For samples near the detection limit, a retest with 2 spots will be relevant.

In conclusion, the newly developed real-time PCR-based assay, Alinity m HBV, can correctly detect HBV DNA in DBS, especially when using the clinically relevant cutoffs of 2,000 IU/mL and 20,000 IU/mL. Validation of existing assays that can be used with DBS to detect and quantify HBV DNA is expected to expand access to hepatitis testing, especially in community settings. Therefore, there is an increasing need for validated standardized manufacturer protocols and registration of quantitative and qualitative HBV molecular diagnostic assays using DBS as a sample type.

## MATERIALS AND METHODS

### Study design.

Plasma specimens were tested using two distinct real-time PCR assays, including the Alinity m HBV (Abbott Molecular, Des Plaines, IL) and Xpert HBV viral load (Cepheid, Sunnyvale, CA) assays, while DBS samples were tested using the Alinity m HBV assay with the Alinity m DBS buffer kit (Abbott Molecular) for elution.

### Clinical specimens.

Paired plasma and DBS samples were obtained from patients recruited in the Department of Hepatology of Hôpital Henri Mondor between January 2018 and May 2019. This retrospective study included 138 patients with chronic HBV infection (defined as the presence of HBsAg more than 6 months, total HBc antibodies, and no anti-HBs). The median age was 43 years, and 71% of patients were male. Hepatitis B virus DNA was detected in 100 HBsAg-positive patients. Genotyping based on phylogenetic analysis of a portion of the overlapping S gene was available for 78 samples, including 14 genotype A, 7 genotype B, 4 genotype C, 10 genotype D, 40 genotype E, and 3 genotype G samples. In the remaining 22 specimens, genotype was not available due to insufficient plasma amount in 2 cases and PCR amplification failure in 20 cases (HBV DNA level < 3 log IU/mL).

Whole-blood samples were collected using a previously described DBS technique (9). Briefly, 50-μL venous whole-blood samples collected in tubes containing EDTA were spotted onto filter paper cards (Whatman 930; GE Healthcare Europe, Freiburg, Germany). The filter paper was then placed onto a clean dry air surface for at least 1 h. Each dried DBS was then stored in an individual sealed plastic bag with a desiccant package at −20°C until analysis. DBS specimens were stored between 22 and 37 months.

The study was conducted in accordance with the principles of good clinical practice and in adherence with the Declaration of Helsinki. Only leftover samples from samples originally sent to the laboratory for routine testing were used. All samples were anonymized before the start of the study. Each leftover sample was assigned an identification number that did not include any patient identifier.

### Laboratory measurements.

Blood samples were screened for HBV markers (HBsAg, anti-HBc, and anti-HBs) by automated immunoassays using the VITROS ECi/ECiQ immunodiagnostic system (Ortho-Clinical Diagnostics, Raritan, NJ). Plasma HBV DNA levels were measured using Xpert HBV viral load or Alinity m HBV assay according to the manufacturer’s instructions. HBV genotypes was determined by direct sequencing of a portion of the overlapping genes encoding HBsAg and the B and C subdomains of the HBV reverse transcriptase as previously described (20).

For DBS samples, a punched disk (12-mm diameter) was eluted in 1.5 mL of a preextraction buffer (mSample preparation system DBS buffer; Abbott Molecular, Des Plaines, IL) for 30 min at room temperature and centrifuged at 36,200 × *g* for 2 min before use (21). A total of 300 μL of preextraction supernatant was added to run the Alinity m HBV assay.

### Statistical analysis.

Descriptive statistics are presented as mean values ± SDs. Relationships between quantitative variables were investigated by Deming or linear regression analysis. The Bland-Altman plot method was used to better visualize differences between quantification assays. Comparisons between groups were performed using the Kruskal-Wallis test or the Mann-Whitney U test. A *p* value of <0.05 was considered statistically significant. Results for DBS samples were not corrected for hematocrit.

## References

[1] Hellard ME, Chou R, Easterbrook P. 2017. WHO guidelines on testing for hepatitis B and C—meeting targets for testing. BMC Infect Dis 17:703. doi:10.1186/s12879-017-2765-2. 29143613 PMC5688468

[2] Polaris Observatory Collaborators. 2018. Global prevalence, treatment, and prevention of hepatitis B virus infection in 2016: a modelling study. Lancet Gastroenterol Hepatol 3:383–403. doi:10.1016/S2468-1253(18)30056-6. 29599078

[3] Hutin Y, Nasrullah M, Easterbrook P, Nguimfack BD, Burrone E, Averhoff F, Bulterys M. 2018. Access to treatment for hepatitis B virus infection—worldwide, 2016. MMWR Morb Mortal Wkly Rep 67:773–777. doi:10.15585/mmwr.mm6728a2. PMC6054001 30025413

[4] European Association for the Study of the Liver. 2017. EASL 2017 Clinical Practice Guidelines on the management of hepatitis B virus infection. J Hepatol 67:370–398. doi:10.1016/j.jhep.2017.03.021. 28427875

[5] Terrault NA, Lok ASF, McMahon BJ, Chang KM, Hwang JP, Jonas MM, Brown RS, Jr, Bzowej NH, Wong JB. 2018. Update on prevention, diagnosis, and treatment of chronic hepatitis B: AASLD 2018 hepatitis B guidance. Hepatology 67:1560–1599. doi:10.1002/hep.29800. 29405329 PMC5975958

[6] Chevaliez S, Dauvillier C, Dubernet F, Poveda JD, Laperche S, Hezode C, Pawlotsky JM. 2017. The new Aptima HBV Quant real-time TMA assay accurately quantifies hepatitis B virus DNA from genotypes A to F. J Clin Microbiol 55:1211–1219. doi:10.1128/JCM.02219-16. 28202793 PMC5377849

[7] Vanhomwegen J, Kwasiborski A, Diop A, Boizeau L, Hoinard D, Vray M, Bercion R, Ndiaye B, Dublineau A, Michiyuki S, Manuguerra JC, Sauvage V, Candotti D, Seck A, Laperche S, Shimakawa Y. 2021. Development and clinical validation of loop-mediated isothermal amplification (LAMP) assay to diagnose high HBV DNA levels in resource-limited settings. Clin Microbiol Infect 27:1858.e9–1858.e15. doi:10.1016/j.cmi.2021.03.014. 33838304

[8] Bargain P, Heslan C, Thibault V, Pronier C. 2020. Combined use of dried blood spot and rapid molecular systems: a robust solution to monitor hepatitis B virus infection with potential for resource-limited countries. J Virol Methods 283:113908. doi:10.1016/j.jviromet.2020.113908. 32522575

[9] Poiteau L, Wlassow M, Hezode C, Pawlotsky JM, Chevaliez S. 2020. Evaluation of the Xpert HBV Viral Load for hepatitis B virus molecular testing. J Clin Virol 129:104481. doi:10.1016/j.jcv.2020.104481. 32512377

[10] Roger S, Lefeuvre C, Grison M, Ducancelle A, Lunel-Fabiani F, Pivert A, Le Guillou-Guillemette H. 2020. Evaluation of the Aptima HBV Quant Dx assay for semi-quantitative HBV viral load from dried blood spots. J Clin Virol 129:104524. doi:10.1016/j.jcv.2020.104524. 32629186

[11] Lange B, Cohn J, Roberts T, Camp J, Chauffour J, Gummadi N, Ishizaki A, Nagarathnam A, Tuaillon E, van de Perre P, Pichler C, Easterbrook P, Denkinger CM. 2017. Diagnostic accuracy of serological diagnosis of hepatitis C and B using dried blood spot samples (DBS): two systematic reviews and meta-analyses. BMC Infect Dis 17:700. doi:10.1186/s12879-017-2777-y. 29143672 PMC5688450

[12] Bonanzinga S, Onelia F, Jackson K, Glass A, Maree L, Krugel M, Pacenti M, Gunson R, Goldstein E, Garcia LM, Galan JC, Vilas A, Ehret R, Knechten H, Naeth G, Braun P, Obermeier M, Marlowe N, Palm MJ, Pfeifer K, Joseph AM, Dhein J, Reinhardt B, Lucic D, Chevaliez S. 2020. Multicenter clinical evaluation of Alinity m HBV assay performance. J Clin Virol 129:104514. doi:10.1016/j.jcv.2020.104514. 32688328

[13] Catlett B, Carrera A, Starr M, Applegate TL, Lowe P, Grebely J, Philip Cunningham H. 2019. Performance evaluation of the Hologic Aptima HCV Quant Dx assay for detection of HCV RNA from dried blood spots. J Clin Virol 112:40–44. doi:10.1016/j.jcv.2019.01.010. 30776575

[14] Marcuccilli F, Chevaliez S, Muller T, Colagrossi L, Abbondanza G, Beyser K, Wlassow M, Ortonne V, Perno CF, Ciotti M. 2021. Multicenter evaluation of the Cepheid Xpert((R)) HBV viral load test. Diagnostics (Basel) 11:297. doi:10.3390/diagnostics11020297. 33673365 PMC7917951

[15] Weber J, Sahoo MK, Taylor N, Uy E, Shi RZ, Pinsky BA. 2019. Comparison of transcription-mediated amplification and real-time PCR assays for hepatitis B virus DNA quantitation in serum. J Appl Lab Med 4:383–390. doi:10.1373/jalm.2019.029058. 31659075

[16] Maasoumy B, Bremer B, Lehmann P, Marins EG, Michel-Treil V, Simon CO, Njoya M, Cornberg M, Paxinos E, Manns MP, Vermehren J, Sarrazin C, Sohn JY, Cho Y, Wedemeyer H. 2017. Commutability and concordance of four hepatitis B virus DNA assays in an international multicenter study. Therap Adv Gastroenterol 10:609–618. doi:10.1177/1756283X17722745. PMC5557192 28835775

[17] Ortonne V, Wlassow M, Bouvier-Alias M, Melica G, Poveda JD, Laperche S, Pawlotsky JM, Chevaliez S. 2021. Diagnosis and monitoring of hepatitis B virus infection using the Cobas((R)) HBV test for use on the Cobas((R)) 4800 system. Microorganisms 9:573. doi:10.3390/microorganisms9030573. 33799562 PMC7999133

[18] Coffin CS, Zhou K, Terrault NA. 2019. New and old biomarkers for diagnosis and management of chronic hepatitis B virus infection. Gastroenterology 156:355–368.e3. doi:10.1053/j.gastro.2018.11.037. 30472225 PMC6433165

[19] Martinez-Camprecios J, Rando-Segura A, Buti M, Rodrigo-Velasquez F, Riveiro-Barciela M, Barreira-Diaz A, Alvarez-Lopez P, Salmeron P, Palom A, Tabernero D, Palomo N, Nindia A, Barbosa G, Lopez E, Ferreira V, Saiago N, Kuchta A, Ferrer-Costa R, Esteban R, Molina I, Rodriguez-Frias F. 2021. Reflex viral load testing in dried blood spots generated by plasma separation card allows the screening and diagnosis of chronic viral hepatitis. J Virol Methods 289:114039. doi:10.1016/j.jviromet.2020.114039. 33338545

[20] Pallier C, Castera L, Soulier A, Hezode C, Nordmann P, Dhumeaux D, Pawlotsky JM. 2006. Dynamics of hepatitis B virus resistance to lamivudine. J Virol 80:643–653. doi:10.1128/JVI.80.2.643-653.2006. 16378967 PMC1346832

[21] Goldstein EJ, Shepherd SJ, Gunson RN. 2020. Investigating utilising the Alinity m platform to detect hepatitis C virus RNA in dried blood spot samples. J Clin Virol 132:104647. doi:10.1016/j.jcv.2020.104647. 32979769

