# Supplementary material for: Alinity m, a Random-Access System, for Hepatitis B Virus DNA Quantification in Plasma and Whole Blood Collected on Dried Blood Spots
Source: mSphere. 2022 Apr 28;7(3):e00082-22. doi: 10.1128/msphere.00082-22 (PMC9241498; doi:10.1128/msphere.00082-22)
Supplement: TABLE S1 [file msphere.00082-22-s0001.docx]

**Table S1:** Sensitivity and specificity of the Alinity m HBV assay for HBV DNA detection in whole blood collected on DBS compared to plasma.

|  |  | **Alinity m HBV (plasma)** | | | |
| --- | --- | --- | --- | --- | --- |
|  |  | | No. of detectable | No. of undetectable | Total |
| **Alinity m HBV (DBS)** | No of detectable | | 75 | 0 | 75 |
|  | No. of undetectable | | 21 | 34 | 54 |
|  | Total | | 96 | 34 | 130 |
